# [1,2-Bis(pyridin-2-ylmeth­oxy)benzene-κ^4^ *N*,*O*,*O*′,*N*′]dichloridocopper(II)

**DOI:** 10.1107/S160053681101333X

**Published:** 2011-04-16

**Authors:** Nan-Nan Huang, Shuang Zhang, Ying Liu, Guang-Feng Hou, Jin-Sheng Gao

**Affiliations:** a Pharmaceutical College, Heilongjiang University of Traditional Chinese Medicine, Harbin 150040, People’s Republic of China; b Engineering Research Center of Pesticides of Heilongjiang Province, Heilongjiang University, Harbin 150080, People’s Republic of China; c College of Chemistry and Materials Science, Heilongjiang University, Harbin 150080, People’s Republic of China

## Abstract

In the title compound, [CuCl_2_(C_18_H_16_N_2_O_2_)], the Cu^II^ atom lies on a twofold axis and is six-coordinated in a distorted octa­hedral environment defined by two N and two O atoms from the ligand and by two Cl atoms. In the crystal, π–π inter­actions [centroid–centroid distance = 3.838 (1) Å] and C—H⋯Cl hydrogen bonds link adjacent mol­ecules into a chain structure along [101].

## Related literature

For related structures, see: Zhang *et al.* (2010*a* ▶,*b* ▶).
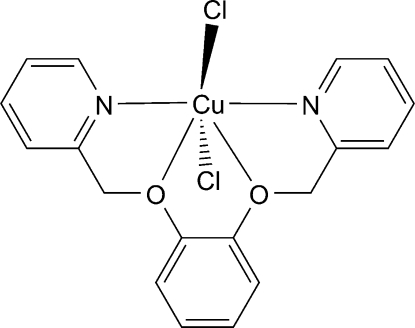


## Experimental

### 

#### Crystal data

[CuCl_2_(C_18_H_16_N_2_O_2_)]
*M* *_r_* = 426.77
Monoclinic, 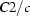

*a* = 10.624 (2) Å
*b* = 19.458 (4) Å
*c* = 8.8063 (18) Å
β = 101.35 (3)°
*V* = 1784.8 (6) Å^3^
*Z* = 4
Mo *K*α radiation
μ = 1.54 mm^−1^
*T* = 293 K
0.21 × 0.19 × 0.16 mm

#### Data collection

Rigaku R-AXIS RAPID diffractometer
Absorption correction: multi-scan (*ABSCOR*; Higashi, 1995 ▶) *T*_min_ = 0.739, *T*_max_ = 0.790
7741 measured reflections
2045 independent reflections
1637 reflections with *I* > 2σ(*I*)
*R*_int_ = 0.035

#### Refinement

*R*[*F* ^2^ > 2σ(*F* ^2^)] = 0.034
*wR*(*F* ^2^) = 0.081
*S* = 1.05
2045 reflections
114 parameters
H-atom parameters constrained
Δρ_max_ = 0.31 e Å^−3^
Δρ_min_ = −0.23 e Å^−3^

### 

Data collection: *RAPID-AUTO* (Rigaku, 1998 ▶); cell refinement: *RAPID-AUTO*; data reduction: *CrystalStructure* (Rigaku/MSC, 2002 ▶); program(s) used to solve structure: *SHELXS97* (Sheldrick, 2008 ▶); program(s) used to refine structure: *SHELXL97* (Sheldrick, 2008 ▶); molecular graphics: *SHELXTL* (Sheldrick, 2008 ▶); software used to prepare material for publication: *SHELXL97*.

## Supplementary Material

Crystal structure: contains datablocks I, global. DOI: 10.1107/S160053681101333X/ng5145sup1.cif

Structure factors: contains datablocks I. DOI: 10.1107/S160053681101333X/ng5145Isup2.hkl

Additional supplementary materials:  crystallographic information; 3D view; checkCIF report

## Figures and Tables

**Table 1:** Hydrogen-bond geometry (Å, °)

*D*—H⋯*A*	*D*—H	H⋯*A*	*D*⋯*A*	*D*—H⋯*A*
C6—H6*A*⋯Cl1^i^	0.97	2.65	3.541 (3)	153

Symmetry code: (i) 
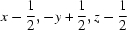
.

**Fig. 1.**
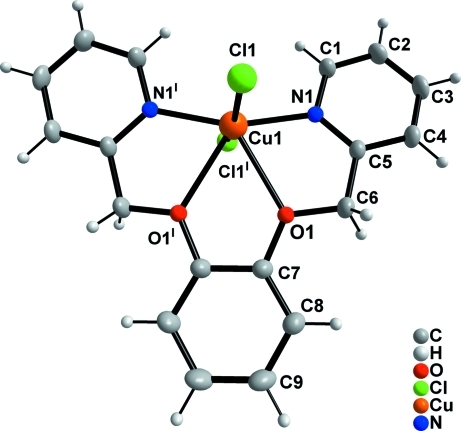
The molecular structure of the title compound, showing displacement ellipsoids at the 50% probability level for non-H atoms. Symmetry code I: 1 - x, y, 1.5 - z.

**Fig. 2.**
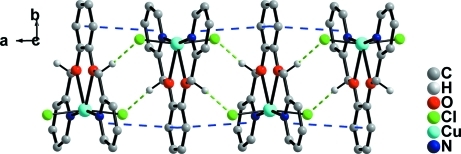
A partial packing view, showing the chain structure. Dashed lines indicate the hydrogen bonds (green) and πi—πi interactions (blue), no involving H atoms have been omitted for clarity.

## supplementary crystallographic information

### Comment

N-heterocyclic ligands coordinated with transition metal ions can form a variety of topology structures, including macrocycles, polyhedra and linear and helical polymers. Our group has report three kinds of flexible pyridyl-based ligands in the previous report. As a part of our continuing work for bipyridyl aromatic ligands, we report the crystal structure of the title compound here.

1,2-Bis(pyridin-2-ylmethoxy)benzene molecule act as a chelating ligand to coordinate with Cu^II^ ion forming a discrete strucutre. Two chlorid counter ions also coordinate to the center Cu^II^ ion, resulting a sxi-coordinated distorted octahedral geometry environment (Figure 1).

In the crystal, the πi—πi interactions with distance about 3.838 (1) Å, and the C—H···Cl hydrogen bonds link these isolated molecules to form a chain structure along [101] direction (Figure 2, Table 1).

### Experimental

The 1,2-Bis(pyridin-2-ylmethoxy)benzene was synthesized by the reaction of ο-dihydroxybenzene and 2-chloromethylpyridine hydrochloride under nitrogen atmosphere and alkaline condition (Zhang *et al.*, 2010*a*). Title ligand (0.58 g, 0.02 mol) and CuCl_2_ (0.27 g, 0.02 mol) were dissolved in 15 mL e thanol, and then the mixture keep stirring for 30 minute. The resulting solution was filtered, and the filtrate was allowed to stand in a desiccator at room temperature for several days. Bule needle crystals were obtained with yield 57%.

### Refinement

H atoms bound to C atoms were placed in calculated positions and treated as riding on their parent atoms, with C—H = 0.93 Å (aromatic C), C—H = 0.97 Å (methene C), and with *U*_iso_(H) = 1.2*U*_eq_(C).

### Crystal data

**Table:**

[CuCl_2_(C_18_H_16_N_2_O_2_)]	*F*(000) = 868
*M**_r_* = 426.77	*D*_x_ = 1.588 Mg m^−^^3^
Monoclinic, *C*2/*c*	Mo *K*α radiation, λ = 0.71073 Å
Hall symbol: -C 2yc	Cell parameters from 6110 reflections
*a* = 10.624 (2) Å	θ = 3.2–27.5°
*b* = 19.458 (4) Å	µ = 1.54 mm^−^^1^
*c* = 8.8063 (18) Å	*T* = 293 K
β = 101.35 (3)°	Block, green
*V* = 1784.8 (6) Å^3^	0.21 × 0.19 × 0.16 mm
*Z* = 4	

### Data collection

**Table:**

Rigaku R-AXIS RAPID diffractometer	2045 independent reflections
Radiation source: fine-focus sealed tube	1637 reflections with *I* > 2σ(*I*)
graphite	*R*_int_ = 0.035
ω scans	θ_max_ = 27.5°, θ_min_ = 3.2°
Absorption correction: multi-scan (*ABSCOR*; Higashi, 1995)	*h* = −13→13
*T*_min_ = 0.739, *T*_max_ = 0.790	*k* = −25→25
7741 measured reflections	*l* = −11→9

### Refinement

**Table:**

Refinement on *F*^2^	Primary atom site location: structure-invariant direct methods
Least-squares matrix: full	Secondary atom site location: difference Fourier map
*R*[*F*^2^ > 2σ(*F*^2^)] = 0.034	Hydrogen site location: inferred from neighbouring sites
*wR*(*F*^2^) = 0.081	H-atom parameters constrained
*S* = 1.05	*w* = 1/[σ^2^(*F*_o_^2^) + (0.0321*P*)^2^ + 1.5862*P*] where *P* = (*F*_o_^2^ + 2*F*_c_^2^)/3
2045 reflections	(Δ/σ)_max_ < 0.001
114 parameters	Δρ_max_ = 0.31 e Å^−^^3^
0 restraints	Δρ_min_ = −0.23 e Å^−^^3^

### Special details

**Table:**

Geometry. All e.s.d.'s (except the e.s.d. in the dihedral angle between two l.s. planes) are estimated using the full covariance matrix. The cell e.s.d.'s are taken into account individually in the estimation of e.s.d.'s in distances, angles and torsion angles; correlations between e.s.d.'s in cell parameters are only used when they are defined by crystal symmetry. An approximate (isotropic) treatment of cell e.s.d.'s is used for estimating e.s.d.'s involving l.s. planes.
Refinement. Refinement of *F*^2^ against ALL reflections. The weighted *R*-factor *wR* and goodness of fit *S* are based on *F*^2^, conventional *R*-factors *R* are based on *F*, with *F* set to zero for negative *F*^2^. The threshold expression of *F*^2^ > σ(*F*^2^) is used only for calculating *R*-factors(gt) *etc*. and is not relevant to the choice of reflections for refinement. *R*-factors based on *F*^2^ are statistically about twice as large as those based on *F*, and *R*- factors based on ALL data will be even larger.

### Fractional atomic coordinates and isotropic or equivalent isotropic displacement parameters (Å^2^)

**Table:**

	*x*	*y*	*z*	*U*_iso_*/*U*_eq_	
C1	0.3928 (2)	0.42692 (13)	0.4854 (3)	0.0455 (6)	
H1	0.4104	0.4597	0.5635	0.055*	
C2	0.3430 (2)	0.44885 (14)	0.3369 (3)	0.0474 (6)	
H2	0.3282	0.4953	0.3152	0.057*	
C3	0.3159 (2)	0.40034 (14)	0.2217 (3)	0.0480 (6)	
H3	0.2825	0.4135	0.1203	0.058*	
C4	0.3387 (2)	0.33231 (13)	0.2581 (2)	0.0431 (6)	
H4	0.3195	0.2988	0.1817	0.052*	
C5	0.3910 (2)	0.31374 (12)	0.4105 (2)	0.0363 (5)	
C6	0.4195 (3)	0.24013 (13)	0.4492 (3)	0.0471 (6)	
H6A	0.3451	0.2122	0.4074	0.057*	
H6B	0.4904	0.2249	0.4031	0.057*	
O1	0.4510 (2)	0.23204 (9)	0.60859 (18)	0.0680 (6)	
C7	0.4747 (2)	0.16783 (12)	0.6701 (3)	0.0445 (6)	
C8	0.4510 (2)	0.10685 (13)	0.5912 (3)	0.0489 (6)	
H8	0.4186	0.1067	0.4851	0.059*	
C9	0.4759 (3)	0.04561 (14)	0.6716 (3)	0.0555 (7)	
H9	0.4600	0.0041	0.6191	0.067*	
Cl1	0.69527 (6)	0.35669 (4)	0.68252 (7)	0.0570 (2)	
Cu1	0.5000	0.34350 (2)	0.7500	0.03802 (14)	
N1	0.41728 (18)	0.36093 (10)	0.52330 (19)	0.0375 (4)	

### Atomic displacement parameters (Å^2^)

**Table:**

	*U*^11^	*U*^22^	*U*^33^	*U*^12^	*U*^13^	*U*^23^
C1	0.0599 (16)	0.0429 (14)	0.0324 (12)	−0.0005 (11)	0.0060 (10)	0.0007 (10)
C2	0.0516 (15)	0.0499 (15)	0.0402 (13)	0.0055 (12)	0.0078 (10)	0.0116 (11)
C3	0.0450 (14)	0.0692 (18)	0.0282 (12)	0.0031 (12)	0.0033 (9)	0.0103 (11)
C4	0.0443 (14)	0.0569 (16)	0.0265 (10)	−0.0026 (11)	0.0032 (9)	−0.0041 (10)
C5	0.0385 (12)	0.0432 (13)	0.0270 (11)	−0.0039 (10)	0.0059 (8)	−0.0025 (9)
C6	0.0662 (16)	0.0438 (14)	0.0292 (11)	−0.0031 (12)	0.0041 (10)	−0.0045 (10)
O1	0.1351 (19)	0.0334 (10)	0.0290 (9)	0.0025 (10)	0.0000 (9)	−0.0006 (7)
C7	0.0616 (16)	0.0345 (13)	0.0387 (12)	−0.0009 (11)	0.0133 (11)	−0.0010 (9)
C8	0.0631 (16)	0.0415 (14)	0.0444 (14)	−0.0022 (12)	0.0164 (11)	−0.0072 (11)
C9	0.0749 (19)	0.0355 (14)	0.0625 (15)	−0.0039 (12)	0.0287 (14)	−0.0074 (11)
Cl1	0.0554 (4)	0.0796 (5)	0.0357 (3)	0.0167 (3)	0.0081 (3)	0.0120 (3)
Cu1	0.0557 (3)	0.0337 (2)	0.02259 (19)	0.000	0.00265 (15)	0.000
N1	0.0463 (11)	0.0392 (11)	0.0254 (9)	−0.0022 (8)	0.0032 (7)	0.0012 (7)

### Geometric parameters (Å, °)

**Table:**

C1—N1	1.339 (3)	C6—H6B	0.9700
C1—C2	1.379 (3)	O1—C7	1.365 (3)
C1—H1	0.9300	O1—Cu1	2.5040 (18)
C2—C3	1.374 (4)	C7—C8	1.373 (3)
C2—H2	0.9300	C7—C7^i^	1.405 (5)
C3—C4	1.372 (4)	C8—C9	1.385 (4)
C3—H3	0.9300	C8—H8	0.9300
C4—C5	1.395 (3)	C9—C9^i^	1.375 (5)
C4—H4	0.9300	C9—H9	0.9300
C5—N1	1.341 (3)	Cl1—Cu1	2.2820 (8)
C5—C6	1.490 (3)	Cu1—N1^i^	2.0451 (18)
C6—O1	1.386 (3)	Cu1—N1	2.0451 (18)
C6—H6A	0.9700	Cu1—Cl1^i^	2.2820 (8)
			
N1—C1—C2	123.5 (2)	C6—O1—Cu1	112.98 (14)
N1—C1—H1	118.2	O1—C7—C8	126.1 (2)
C2—C1—H1	118.2	O1—C7—C7^i^	113.68 (12)
C3—C2—C1	118.2 (2)	C8—C7—C7^i^	120.18 (15)
C3—C2—H2	120.9	C7—C8—C9	119.2 (2)
C1—C2—H2	120.9	C7—C8—H8	120.4
C4—C3—C2	119.3 (2)	C9—C8—H8	120.4
C4—C3—H3	120.4	C9^i^—C9—C8	120.63 (15)
C2—C3—H3	120.4	C9^i^—C9—H9	119.7
C3—C4—C5	119.5 (2)	C8—C9—H9	119.7
C3—C4—H4	120.2	N1^i^—Cu1—N1	160.91 (11)
C5—C4—H4	120.2	N1^i^—Cu1—Cl1	89.86 (6)
N1—C5—C4	121.4 (2)	N1—Cu1—Cl1	88.01 (6)
N1—C5—C6	119.02 (19)	N1^i^—Cu1—Cl1^i^	88.01 (6)
C4—C5—C6	119.6 (2)	N1—Cu1—Cl1^i^	89.86 (6)
O1—C6—C5	109.80 (19)	Cl1—Cu1—Cl1^i^	167.08 (4)
O1—C6—H6A	109.7	N1^i^—Cu1—O1	129.53 (7)
C5—C6—H6A	109.7	N1—Cu1—O1	69.56 (7)
O1—C6—H6B	109.7	Cl1—Cu1—O1	94.51 (6)
C5—C6—H6B	109.7	Cl1^i^—Cu1—O1	96.67 (6)
H6A—C6—H6B	108.2	C1—N1—C5	118.03 (19)
C7—O1—C6	119.62 (18)	C1—N1—Cu1	115.29 (15)
C7—O1—Cu1	126.26 (14)	C5—N1—Cu1	126.61 (16)

Symmetry codes: (i) −*x*+1, *y*, −*z*+3/2.

### Hydrogen-bond geometry (Å, °)

**Table:**

*D*—H···*A*	*D*—H	H···*A*	*D*···*A*	*D*—H···*A*
C6—H6A···Cl1^ii^	0.97	2.65	3.541 (3)	153.

Symmetry codes: (ii) *x*−1/2, −*y*+1/2, *z*−1/2.
